# Greenhouse gases emissions in rivers of the Tibetan Plateau

**DOI:** 10.1038/s41598-017-16552-6

**Published:** 2017-11-29

**Authors:** Bin Qu, Kelly Sue Aho, Chaoliu Li, Shichang Kang, Mika Sillanpää, Fangping Yan, Peter A. Raymond

**Affiliations:** 1 Yale-NUIST Center on Atmospheric Environment, International Joint Laboratory on Climate and Environment Change (ILCEC), Nanjing University of Information Science and Technology, Nanjing, 210044 China; 2 Laboratory of Green Chemistry, Lappeenranta University of Technology, Mikkeli, 50130 Finland; 3 Key Laboratory of Tibetan Environment Changes and Land Surface Processes, Institute of Tibetan Plateau Research, Chinese Academy of Sciences, Beijing, 100101 China; 4 Yale School of Forestry and Environmental Studies, Yale University, New Haven, Connecticut 06405 USA; 5 CAS Center for Excellence in Tibetan Plateau Earth Sciences, Chinese Academy of Sciences, Beijing, 100085 China; 6 State Key Laboratory of Cryospheric Science, Northwest Institute of Eco-Environmental and resources, Chinese Academy of Sciences, Lanzhou, Gansu 730000 China; 7 University of Chinese Academy of Sciences, Beijing, 100049 China; 8 Department of Civil and Environmental Engineering, Florida International University, Miami, FL-33174 USA

## Abstract

Greenhouse gases (GHGs) emissions from streams are important to regional biogeochemical budgets. This study is one of the first to incorporate stream GHGs (CO_2_, CH_4_ and N_2_O) concentrations and emissions in rivers of the Tibetan Plateau. With one-time sampling from 32 sites in rivers of the plateau, we found that most of the rivers were supersaturated with CO_2_, CH_4_ and N_2_O during the study period. Medians of partial pressures of CO_2_ (pCO_2_), pCH_4_ and pN_2_O were presented 864 μatm, 6.3 μatm, and 0.25 μatm respectively. Based on a scaling model of the flux of gas, the calculated fluxes of CO_2_, CH_4_ and N_2_O (3,452 mg-C m^2^ d^−1^, 26.7 mg-C m^2^ d^−1^ and 0.18 mg-N m^2^ d^−1^, respectively) in rivers of the Tibetan Plateau were found comparable with most other rivers in the world; and it was revealed that the evasion rates of CO_2_ and CH_4_ in tributaries of the rivers of the plateau were higher than those in the mainstream despite its high altitude. Furthermore, concentrations of GHGs in the studied rivers were related to dissolved carbon and nitrogen, indicating that riverine dissolved components could be used to scale GHGs envision in rivers of the Tibetan Plateau.

## Introduction

The input of carbon (C) and nitrogen (N) from land to water leads most rivers in the world to be supersaturated with greenhouse gases (GHGs, i.e. CO_2_, CH_4_ and N_2_O) and therefore net sources of GHGs to the atmosphere^1–5^. Rivers are reactors for degradation and metabolic processes among aqueous C and N, making them active areas of GHGs with the atmosphere^2,6–10^. For example, after entering into the aquatic system from the land and atmosphere, part of the organic carbon will undergo degradation and result in GHGs emissions^2,9–12^. At the same time, denitrification and nitrification in aquatic system will also alter the nitrogen pools and emit N_2_O gas, which has a global warming potential approximately 265 times that of CO_2_, to the atmosphere^13–15^. It was estimated that CO_2_ emissions from global streams are at 1.8 × 10^6^ Gg C d^−1^ ^3^, while the size of inland water CH_4_ and N_2_O evasion were estimated at 0.2 Gg C d^−1^ and 32.2 Gg N d^−1^, respectively^4,16,17^.

**Figure 1:**
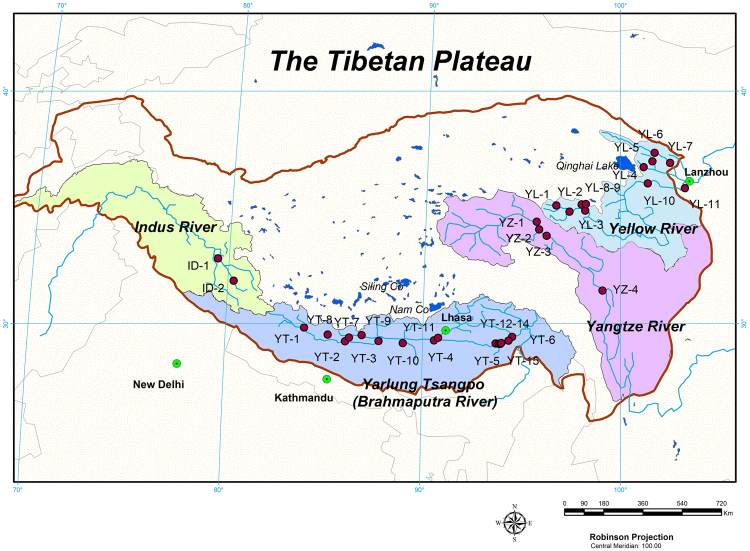
Sampling sites for greenhouse gases (GHGs) in rivers of the Tibetan Plateau. Note: the Indus River is abbreviated to “ID”, the Yarlung Tsangpo is to “YT”, the Yangtze River is to “YZ” and the Yellow River is to “YL”; information on selected rivers and detailed data are shown in Table S1. Hydrographical data of the drainage area are from^26^ and the map was generated by ArcGIS 10.2 (ESRI Inc.).

There are more than ten large rivers in Asia originating from the Tibetan Plateau that provide water resources for billions of people^18^. With climate change and increasing anthropogenic activities, changes in global riverine C and N have been suggested during the last decades^19–21^, including rivers on the Tibetan Plateau^22,23^. It was reported that concentrations of dissolved inorganic carbon (DIC) are elevated in river basins of the plateau, due to the extensive topographic relief and soil erosion^24^. In addition, though concentrations of dissolved organic carbon (DOC) were low, average concentrations of riverine nitrogen on the Tibetan Plateau were close to the world’s mean level, which leads to low DOC/DON ratios (C/N)^24^. Low C/N in rivers usually means that the dissolved organic matters are more bioavailable and more easily decomposed into GHGs emissions^25^. Therefore, along with climate change and increasing anthropogenic activities on the Tibetan Plateau, a growing export of bioavailable riverine carbon and nitrogen can be expected^24^, which will possibly influence GHGs emissions in rivers of the plateau. Furthermore, compared with rivers in other regions of the world, rivers on the Tibetan Plateau have large slopes, due to their huge drop in topography^26^. Large slopes of the river catchments usually lead to large stream velocity, which will result in a high gas transfer velocity (k)^27^. Nevertheless, few literatures have been documented on the Tibetan Plateau, despite their potential interaction with the GHGs budgets in this critical region^13,14,28^. Here we present data from four major watersheds (the Indus, the Yarlung Tsangpo, the Yangtze River and the Yellow River, Fig. 1) on the Tibetan Plateau to 1) assess the spatial distributions of GHGs (CO_2_, CH_4_ and N_2_O) concentrations across the rivers, and 2) investigate the potential factors (e.g., dissolved riverine carbon and nitrogen matter, water temperature, precipitation and land cover types, etc.) that affect GHGs emissions from rivers in this ecological-fragile region.

## Results

### Spatial distribution of pGHGs in the rivers of the Tibetan Plateau

**Figure 2:**
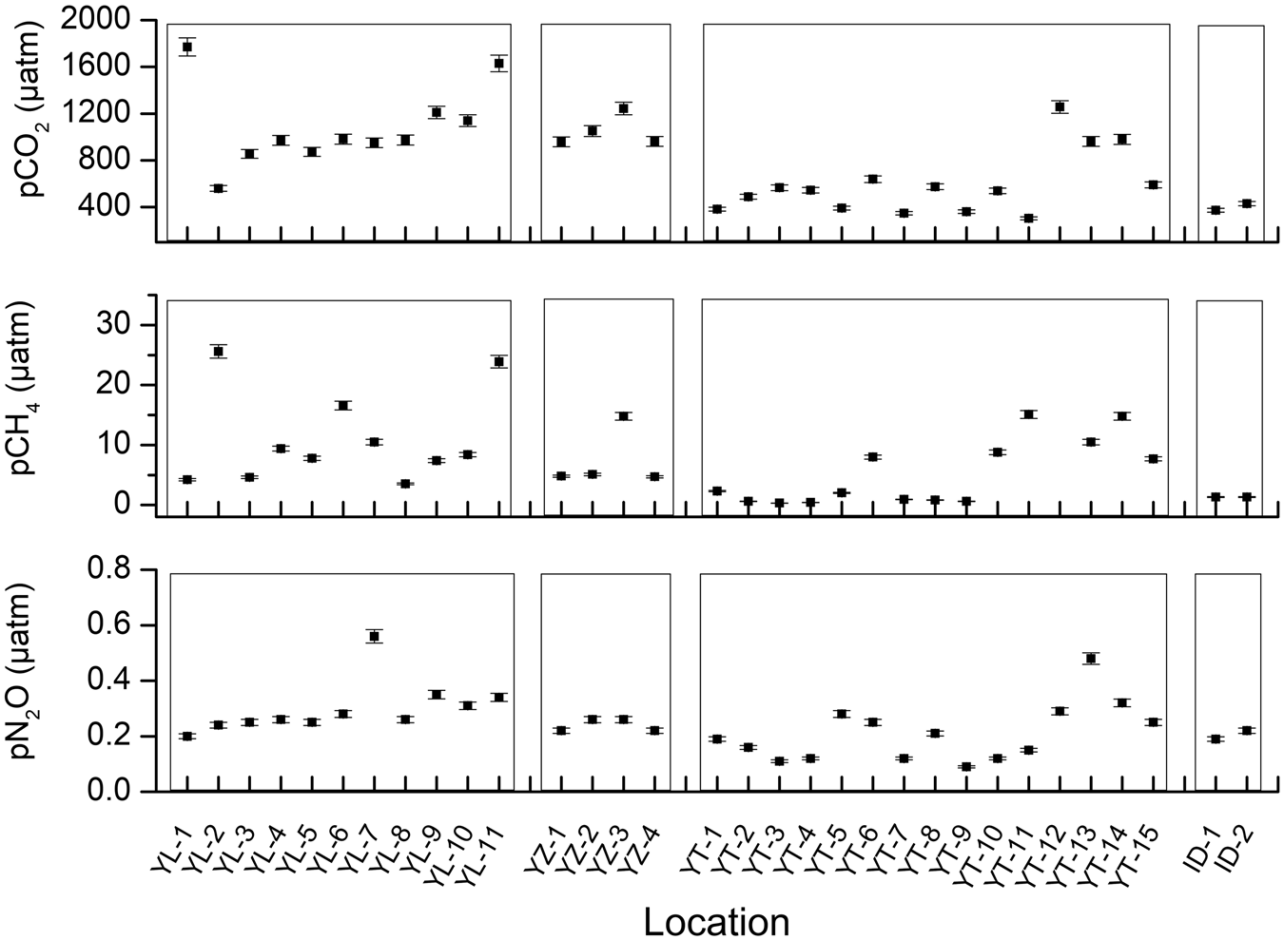
Partial pressures of GHGs distribution in rivers of the Tibetan Plateau. “YL”, “YZ”, “YT” and “ID” are presented for sampling sites in the Yellow River, the Yangtze River, the Yarlung Tsangpo and Indus, respectively. The figure was plotted by Origin 8.5 (Originlab Corp.).

Partial pressures of GHGs (pGHGs, including pCO_2_, pCH_4_ and pN_2_O) in the waters and atmosphere of the studied rivers showed that most of the sampling sites in the watersheds were supersaturated with CO_2_, CH_4_ and N_2_O during the sampling period (Table S3). The pGHGs on the Tibetan Plateau varied largely within the different river basins. For instance, the maximum and minimum pCO_2_ of the Yellow River was 1771 μatm and 560 μatm, respectively (Fig. 2). Similarly, two end values of pCH_4_ in Yarlung Tsangpo were 0.3 μatm and 200.5 μatm. To avoid the defect of such abnormal extreme values, medians for the pGHGs instead of averages were employed in this study in order to discuss the GHGs in rivers of the Tibetan Plateau.

The pCO_2_ on the Tibetan Plateau ranged from 304 μatm to 1771 μatm with a median of 864 μatm, pCH_4_ ranged from 0.3 to 200.5 μatm with a median of 6.3 μatm, and pN_2_O ranged from 0.09 to 0.56 with a median of 0.25 μatm. The Yellow River and the Yangtze River had higher pGHGs than those in the Yarlung Tsangpo and the Indus (Fig. 2). pCO_2_ in rivers of the Tibetan Plateau were lower than the other streams in the world. For instance, concentrations of CO_2_ in rivers of Alaska, Canada and conterminous U.S. ranged from 570–3100 μatm^2,29,30^, while pCO_2_ in Amazon was as high as ~4000 μatm, four times higher than that in rivers of the Tibetan Plateau^31^. CH_4_ concentrations in rivers of the Tibetan Plateau were similar with streams in Alaska^30^, but much lower than those waters in fens or marsh (>40) in the boreal area^32^. Low pGHGs in rivers of the Tibetan Plateau may be mainly due to its high altitude (low atmospheric pressure) and low concentrations of nutrients. It was observed that pGHG in tributaries was higher than those in mainstreams. This means that streams in the lower order have a higher GHGs emission rate to the atmosphere, which is consistent with previous studies^3^. For instance, partial pressures of CO_2_ in the small tributaries of Yarlung Tsangpo (YT-7, 8 and YT-12~15) ranged from 347 to 1257 μatm with a median of 777 μatm, while pCO_2_ in the mainstream ranged from 304–639 with a lower median of 489 μatm (Fig. 2). Similarly, pCH_4_ in tributaries of the Yarlung Tsangpo were also higher than that of the mainstream (~6 times). We infer that high pCH_4_ in tributaries corresponded with the complex wetland area with reductive conditions in the southeast part of the river basin^33^.

Identifying N_2_O emissions is important for evaluating climate change scenarios and assessing mitigation options^34^. Hu *et al*. (2016) calculated that N_2_O emissions from the global rivers is 32.2 Gg N d^−1^ ^17^, and it was proposed that N_2_O emissions from rivers were still increasing as a result of human activities on land, thereby enhancing nitrogen export to aquatic systems^5^. However, during the sampling period in this study, in-stream pN_2_O was lower than that in the atmosphere in sites YT-2, 3, 4 and YT- 7,8,9, indicating that this area of Yanglung Tsangpo on the Tibetan Plateau might act as a sink of N_2_O rather than the source. Despite the sites mentioned above with regard to acting as a N_2_O sink, it was also found that rivers in the western and southern Tibetan Plateau (the Indus and the Yarlung Tsangpo) presented lower N_2_O concentrations than rivers of the eastern and northern plateau. Partial pressures of N_2_O in small tributaries on the Tibetan Plateau were from 0.09 to 0.48 μatm with a median of 0.25 μatm, also higher than those in the mainstream (0.22 μatm). Different with that of CO_2_ and CH_4_, concentrations of N_2_O on the Tibetan Plateau were similar to most other pristine rivers in the world and much lower than those under intensive anthropogenic activities^35,36^.

### Flux of GHGs in the rivers of the Tibetan Plateau

All streams in the studied river basins on the Tibetan Plateau showed sources of CO_2_ and CH_4_ to the atmosphere during the sampling season. Among them, fluxes of CO_2_ and CH_4_ in the Yellow River (median ~6,333 mg CO_2_-C m^−2^ d^−1^, 48.2 mg CH_4_-C m^−2^ d^−1^) were significantly higher than those in the Yangtze River (median ~3,276 mg CO_2_-C m^−2^ d^−1^, 13.6 mg CH_4_-C m^−2^ d^−1^), the Yarlung Tsangpo (~2,442 mg CO_2_-C m^−2^ d^−1^, 11.9 mg CH_4_-C m^−2^ d^−1^) and the Indus (~2,085 g CO_2_-C m^−2^ d^−1^, 2.4 mg CH_4_-C m^−2^ d^−1^), while the flux of N_2_O in the Yarlung Tsangpo was only 0.07 mg N_2_O-N m^−2^ d^−1^ – much lower than that in the other three rivers (Table S3). Emission rates of both CO_2_ and CH_4_ generally illustrated a decreased trend with an increasing stream order, whereas N_2_O presented an opposite trend. We found first-order streams had high CO_2_ and CH_4_ fluxes relative to the second and third-order stream sites on the Tibetan Plateau. For example, in YL-1 and YL-11 – two small branches of the Yellow River – the CO_2_ fluxes were more than 7,000 mg CO_2_-C m^−2^ d^−1^, which were almost three times higher than those in the Amazon basin (Table 1). Nevertheless, due to steep slopes and high stream velocities, GHGs transfer velocities at some sites of the rivers (e.g., YL-1, 4, 9, YT-8, and so on) are fairly high (Table S2), despite its high altitude and low atmospheric pressure. The flux of CO_2_ and CH_4_ from stream surfaces across the four catchments during the study period had a large range (619–14,260 mg CO_2_-C m^−2^ d^−1^ and −6.0–817.9 mg CH_4_-C m^−2^ d^−1^, respectively). However, the median values were a bit lower than those of global streams^4^. Although N_2_O fluxes in the Tibetan rivers (−2.46–4.8 mg N_2_O-N m^−2^ d^−1^) were small relative to those of CO_2_ and CH_4_ (Tables 1 and 2), notably, N_2_O emission rates of these rivers are similar to those of many other rivers in the world (Table 3), despite the pristine aquatic environment.

**Table 1:** CO_2_ flux estimates from rivers on the Tibetan Plateau and other river basins in the world (mg-C m^−2^ d^−1^).

Water Type (Location)	Estimated flux
Stream (Yellow River, Tibetan Plateau, China)	6,333
Stream (Yangtze River, Tibetan Plateau, China)	3,276
Stream (Yarlung Tsangpo, Tibetan Plateau, China)	2,442
Stream (Indus, Tibetan Plateau, China)	2,085
Stream (interior Alaska)^30^	5,400
Stream (Northern, Sweden)^52^	7,679
Stream (Finland)^53^	975
Small stream (Ontario, Canada)^54^	1,079
Small stream (Quebec, Canada)^29^	3,121
Small stream (Sweden)^55^	8,279
Headwater Stream (conterminous U.S.)^2^	2,844
Stream (Amazon basin)^31^	2,268
Stream (Mississippi)^56^	3,241
Stream (Mid and downstream of Yangtze River, China)^57^	3,551
Stream (Xinjiang river, China)^58^	3,277
Stream (Temperate zone)^2^	6,493

**Table 2:** CH_4_ flux estimates from rivers on the Tibetan Plateau and other water bodies in the world (mg-C m^−2^ d^−1^).

Water Type (Location)	Estimated flux
Streams (Yellow River, Tibetan Plateau, China)	48.2
Streams (Yangtze River, Tibetan Plateau, China)	13.6
Streams (Yarlung Tsangpo, Tibetan Plateau, China)	11.9
Stream (Indus, Tibetan Plateau, China)	2.4
Stream (interior Alaska)^30^	7.7
Beaver pond (Manitoba, Canada)^59^	80.8
Alpine Fen (Alaska)^32^	217.8
Marsh (Alaska)^32^	79.7
Poor fens (interior Alaska)^60^	134.8
Reservoir (Finland)^61^	26.0
Rich fen (control treatment, interior Alaska)^62^	73.7
Stream in peatland (Scotland)^63^	176.2
Stream (Ontario, Canada)^64^	134.8
Stream (Tennessee, U.S.A.)^65^	9.9

**Table 3:** N_2_O flux estimates from rivers on the Tibetan Plateau and other water bodies and river basins in the world (mg-N m^−2^ d^−1^).

Water Type (Location)	Estimated flux
Streams (Yellow River, Tibetan Plateau, China)	0.34
Streams (Yangtze River, Tibetan Plateau, China)	0.18
Streams (Yarlung Tsangpo, Tibetan Plateau, China)	0.07
Stream (Indus, Tibetan Plateau, China)	0.13
Stream (Neuse River, North Carolina, U.S.A.)^66^	0.36
Stream (Hudson River, U.S.A)^67^	0.16
Stream (Southeast China)^35^	0.76–9.51
Stream (Amazon basin)^68^	0.27
River estuary (Tamar, England)^69^	0.27
River estuary (Yangtze River, China)^36^	1.64
Lakes (Finland)^61^	−0.005–0.008

## Discussion

### Potential factors affect the delivery of GHGs in rivers of the Tibetan Plateau

Relationships between the GHGs and other dissolved components in waters, such as DIC/N and DOC/N, added to environmental variables such as pH and water temperature, were explored in order to investigate the potential factors that affect GHGs in the rivers of the Tibetan Plateau (Table 4). These relationships likely result from a joint influence from the chosen index and changes with the equilibrium distribution of the air-water system.

**Table 4:** Pearson correlation (r^2^) between pGHGs (μatm) and dissolved carbon and nitrogen (mg L^−1^) and meteorological (water temperature (°C) and average annual precipitation (mm)).

	DIC (n = 10)	DOC (n = 10)	Temp. (n = 30)	Prep. (n = 10)	pH (n = 22)
pCO_2_	0.76**	0.02	0.01	0.21	0.30 (−)**
	DIC (n = 10)	DOC (n = 10)	Temp. (n = 30)	Prep. (n = 10)	
pCH_4_	0.79**	0.27	0.01	0.05	
	DIN (n = 10)	DON (n = 10)	Temp. (n = 10)	Prep. (n = 10)	
pN_2_O	0.23	0.05	<0.01	0.01 (−)	

Detailed data are presented in Tables S1, S2 and S3. Note: *means correlation at 0.05 (2-tailed); **means correlation at 0.01(2-tailed); (−) means negative correlation.

It was estimated that ~60% DIC exported in York River was lost as CO_2_ evasion to the atmosphere^10^. Partial pressure of CO_2_ in streams of the Tibetan Plateau appears to be significantly correlated with DIC (r^2^ > 0.76, 0.05) within the catchment (Table 4). In addition to being correlated with dissolved carbon matter, pCO_2_ is also correlated with precipitation (Table 4). Precipitation events mainly control in-stream gas concentrations in two ways: (1) increasing gas concentrations by flushing inorganic and organic carbon from the landscape into streams, and (2) decreasing gas concentrations by diluting stream water and facilitating gas exchange^37^. Interestingly, the correlation between the precipitation and pCO_2_ is stronger than that with DOC concentrations and water temperature. We infer that there are two factors which possibly are responsible for the strong correlation between precipitation and CO_2_ concentrations. First, rates of precipitation usually correlate with terrestrial ecosystem fluxes such as annual net primary production^38^ and secondly, the higher annual precipitation usually leads to higher flushing and delivery of soil and riparian/wetland CO_2_ to streams and river^2^. Therefore, we propose that precipitation impacts stream CO_2_ evasion not only on long seasonal timescales but also on short time scales associated with CO_2_ production and flushing processes. A negative relationship between pCO_2_ and pH is expected, as dissolved CO_2_ acts as an acid in water and poorly buffered systems^30^. Moreover, pH can be a strong indicator of the dissolved CO_2_ in the stream^39^.

Similar to pCO_2_, partial pressures of CH_4_ in rivers of the Tibetan Plateau were also elevated with dissolved carbon. Concentrations of dissolved carbon, including DIC and DOC, explained most of the variability in pCH_4_ (Table 4). As a product of anaerobic decomposition of organic matter, there was a positive trend (Table 4) in CH_4_ concentrations with the increased DOC concentrations in the rivers of the Tibetan Plateau, indicating that water temperature placed a certain influence on driving pCH_4_ increased in anaerobic decomposition in these Tibetan rivers. Partial pressures of N_2_O were correlated with dissolved nitrogen (DIN and DON, Table 4). Anthropogenic activities have been important sources of dissolved nitrogen to rivers for several centuries^40^, and it was estimated that more than 90% of the current N_2_O emissions from rivers and estuaries in the world can be considered anthropogenic sources^13^. Most of the large cities on the Tibetan Plateau were located along the river catchment. Therefore, with growing anthropogenic activities such as urbanization, industrial and agricultural activities, more nitrogen substances will enter the mainstream of the rivers than enter the tributaries. This may also explain why the N_2_O concentrations in mainstreams of the Tibetan rivers are higher than those in tributaries.

This is one of the first studies to incorporate stream GHGs (CO_2_, CH_4_ and N_2_O) concentrations and emissions in four large river basins on the Tibetan Plateau, where the most important permafrost area in the mid-latitude region is distributed. Despite the defect that the results are based on single sampling at each observation site in the summer half year of the plateau, we found that most waters in the studied rivers of the Tibetan Plateau were consistently supersaturated with GHGs (CO_2_, CH_4_ and N_2_O) during the sampling season. In-stream pGHGs on the Tibetan Plateau ranged from 300 to 1,800 μatm with pCO_2_ median of 864 μatm, pCH_4_ of 6.3 μatm and pN_2_O of 0.25 μatm, respectively. Concentrations of GHGs in the rivers showed a different spatial pattern across the plateau. In-stream pCO_2_ and pCH_4_ in the Yellow River and the Yangtze River were over two times higher than those in Yarlung Tsangpo and Indus, while the pN_2_O in the Yellow River was the lowest. It was observed that CH_4_ and N_2_O concentrations were one and two orders of magnitude lower than that of CO_2_, respectively. Evasion rates of CO_2_ and CH_4_ in the tributaries were at least two times higher than those in the mainstream of the rivers. The concentrations of GHGs in waters of the Tibetan Plateau rivers were related to dissolved carbon and nitrogen matter, indicating that compared with climatic conditions (i.e., water temperature, precipitation) that could influence the aqueous pGHGs, riverine dissolved components are the key drivers that control the GHGs envision in rivers of this region. Due to high GHGs transfer velocity (k) in rivers of the Tibetan Plateau, the flux of GHGs (~660–14,300 mg-C and N m^2^ d^−1^) presented in a manner similar to many other streams in the world, despite its high altitude and pristine river water environment on the plateau. Nevertheless, with growing agricultural and industrial activities in this ecological-fragile region, a large amount of nutrients will be transported to the aquatic system and increasing GHGs emissions from the rivers can be anticipated. Further studies based on intensive observations (e.g., monthly, seasonally and annually) are needed to identify the full roles of the rivers act in GHGs emissions of the Tibetan Plateau.

## Materials and Methods

### Sampling information and studied river basins

The sampling work was conducted one-time at each observation site with triple parallel samples during the year of 2014 and 2015. The detailed sampling information are listed in Table S1. The studied river basins are 1) The Indus, one of the largest river systems (3,180 km) draining the Himalaya and running across West China, Pakistan and North India^41^; 2) the Yarlung Tsangpo – the upper reach of the Brahmaputra River, running across the South Tibetan Plateau, India and Bangladesh^42^; 3) the Yangtze River, with a drainage area of 1.80 × 10^6^ km^2^ in Euro-Asian Continent, the third longest (6,300 km) and fourth for freshwater flow (900 × 10^9^ m^3^ d^−1^) in the world^43^; 4) the Yellow River, the second largest river (5,464 km) which runs across China^44^. On the plateau region, the drainage basins of the Yarlung Tsangpo and the Yangtze River are larger than those of the Indus River and the Yellow River (Fig. 1). These two rivers are prominently influenced by the India/South Asia monsoon and receive more precipitation in the summer^45^. The four river catchments cover almost half of the Tibetan Plateau (Fig. 1) and have variable meteorological and landscape characteristics^26^. Besides gas samples collected from the mainstreams of the headwater of the four rivers, gases from the mainstem and small tributaries of the Yarlung Tsangpo and the Yellow River were also collected. Three stream orders were defined, based on the joint level of the tributaries with the main river stream. Order 3 stands for the main stem of the river, while order 2 stands for the main tributaries that join to the main stem of order 3. Order 1 stands for the small headwater tributaries that join to the main tributaries in order 2 (Table S1). The hydrological conditions (e.g., discharge, stream velocity, etc.) of the studied rivers were listed in Table S2.

### Method of GHGs & water samples collection and measurement

We used the headspace equilibration method^1^ to collect dissolved greenhouse gas (GHGs) in the studied rivers. Using this method, 40 ml of stream water was equilibrated with 20 ml of ambient air by shaking for 2 minutes underwater to maintain constant temperature. 15 mL of the equilibrated headspace was then sub-sampled and stored in air-tight Exetainer vials. Samples were taken at all study sites in duplicate or triplicate for quality control. Gas concentrations were analyzed using a Shimadzu gas chromatograph (GC-2014) with a flame ionization detector and electron capture detector at the Yale Analytical and Stable Isotope Center.

Water samples for dissolved riverine carbon and nitrogen were collected at approximately 10 cm depth below the surface and filtered with 0.7 μm glass fiber filters in the field. Samples for DIC measurement were stored at room temperature in 500-ml gas-tight brown glass bottles and preserved with 100 μl HgCl_2_ to avoid photic or biological degradation, while DOC and dissolved nitrogen samples were stored in 500-ml acid-cleaned polypropylene bottles and stored in containers at −18 °C until laboratory measurement^46,47^. Concentrations of dissolved inorganic nitrogen (DIN, including NO_3_^−^, NH_4_^+^ and NO_2_^−^) in the water were detected by (HPLC) Dionex ICS 2000 and Dionex ICS 2500. DIC (comprising HCO_3_^−^, CO_3_^2−^ and CO_2_), DOC, and total dissolved nitrogen (TDN) were measured with a TOC analyzer (SHIMADZU-TOC-VCPH).

### GHGs concentration and flux calculation

The initial calculation of the equilibrated headspace concentration of trace gases measured by GC was in units of ppmv. We calculated the trace GHG concentrations in the stream water prior to equilibration by using the law of conservation of mass, Henry’s Law, and the Ideal Gas Law. Henry’s Law can be written as:

1 $${{\rm{k}}}_{{\rm{H}}}={{\rm{C}}}_{{\rm{aq}}}/{{\rm{P}}}_{{\rm{gas}}}$$

where k_H_ is a temperature dependence constant^48^, C_aq_ is the gas concentration in the aqueous solution in mol/L, and P_gas_ is the gas concentration in the air in units of atm. K_H_ therefore has units of mol/L*atm^−1^.

The ideal gas law is written as:

2 $${\rm{PV}}={\rm{nRT}}$$

where P is the pressure of the gas, V is the volume of the gas, n is the amount of the gas measured in moles, T is the temperature in Kelvin, and R is the universal gas constant-equal to 0.08206 L*atm*mol^−1^*K^−1^.

Stream water gas concentrations prior to equilibration were calculated as follows:

3 $${{\rm{C}}}_{{\rm{gasI}}}{{\rm{V}}}_{{\rm{gas}}}+{{\rm{C}}}_{{\rm{aqI}}}{\rm{Vaq}}={{\rm{C}}}_{{\rm{gasF}}}{{\rm{V}}}_{{\rm{gas}}}+{{\rm{C}}}_{{\rm{aqF}}}{{\rm{V}}}_{{\rm{aq}}}$$

I: initial, F: final, solving for C_aqF_ is:

4 $${{\rm{P}}}_{{\rm{gasF}}}{{\rm{k}}}_{{\rm{H}}}={{\rm{C}}}_{{\rm{aqF}}}$$

The initial concentration of gas in the steam (C_aqI_) was found by substituting in Henry’s Law and inputting measured values from the gas chromatograph.

Equations (3) and (4) can be reorganized and combined to yield:

5 $${{\rm{C}}}_{{\rm{aqI}}}=({{\rm{C}}}_{{\rm{gasF}}}-{{\rm{C}}}_{{\rm{gasI}}})({{\rm{V}}}_{{\rm{gas}}}/{{\rm{V}}}_{{\rm{aq}}})+{{\rm{P}}}_{{\rm{gasF}}}{{\rm{k}}}_{{\rm{H}}{\rm{.}}}$$

We used the ideal gas law to convert C_gasI_ and C_gasF_ from units of ppmv to mol/L, using the temperature of the water, estimated atmospheric pressure, and R. Substituting in constants and calculated values into equation (3) allowed us to solve for the concentration of dissolved GHGs in the water (C_aqI_).

Flux rates of trace GHGs were calculated using the equation:

6 $${\rm{Flux}}=k\ast ({[{{\rm{CO}}}_{2}]}_{{\rm{aq}}}-{[{{\rm{CO}}}_{2}]}_{{\rm{sat}}})$$

where k is the gas transfer velocity and ([CO_2_]_aq  _− [CO_2_]_sat_) is the concentration gradient between the actual CO_2_ concentration that would be present when CO_2_ is in equilibrium with the atmosphere^49^. [CO_2_]_sat_ is found by multiplying the current partial pressure of (pCO_2_)^50^ in μatm by k_H_.

Gas transfer velocity was estimated with the models from Raymond *et al*. (2012), which are not regional estimates and have been used against other methods^51^. With the caution against the use of these models when attempting to perform process-based studies, we employed the one below (equation 7) to conduct the k calculation in rivers of the Tibetan Plateau.

7 $${\rm{k}}={\rm{VS}}\times 2841\pm 107+2.02\pm 0.209$$

where V is stream velocity (m s^−1^) and S is slope (unitless)^27^.

## Electronic supplementary material

Supplementary Information
