# Supplementary material for: Greenhouse gases emissions in rivers of the Tibetan Plateau
Source: Sci Rep. 2017 Nov 29;7:16573. doi: 10.1038/s41598-017-16552-6 (PMC5707396; doi:10.1038/s41598-017-16552-6)
Supplement: Supplementary file 1 — Supplementary Information [file 41598_2017_16552_MOESM1_ESM.pdf]

Supplementary information

Greenhouse gases emissions in rivers of the Tibetan Plateau

Bin Qu[1,2,3*], Kelly Sue Aho[4], Chaoliu Li[2,3,5*], Shichang Kang[5,6,7], Mika Sillanpää[2,8], Fangping Yan[2], Peter A. Raymond[4]

[1] Yale-NUIST Center on Atmospheric Environment, International Joint Laboratory on Climate and Environment Change (ILCEC), Nanjing University of Information Science & Technology, Nanjing 210044, China

[2] Laboratory of Green Chemistry, Lappeenranta University of Technology, Mikkeli 50130, Finland

[3] Key Laboratory of Tibetan Environment Changes and Land Surface Processes, Institute of Tibetan Plateau Research, Chinese Academy of Sciences, Beijing 100101, China

[4] Yale School of Forestry and Environmental Studies, Yale University, New Haven, Connecticut 06405, USA

[5] CAS Center for Excellence in Tibetan Plateau Earth Sciences, Chinese Academy of Sciences, Beijing 100085, China

[6] State Key Laboratory of Cryospheric Science, Northwest Institute of Eco-Environmental and resources, Chinese Academy of Sciences, Lanzhou, Gansu 730000, China

[7] University of Chinese Academy of Sciences, Beijing 100049, China

[8] Department of Civil and Environmental Engineering, Florida International University, Miami, FL-33174, USA

*Email:

Bin Qu bin.b.qu@outlook.com;

Chaoliu Li lichaoliu@itpcas.ac.cn

Contents

1. Supporting tables

Table S1. Sampling information in rivers of the Tibetan Plateau.

Table S2. Estimated k (m d^{-1}) based on stream velocity (V, in m s^{-1}) and slope (S; unitless) for the sampling sites.

Table S3. GHGs partial pressures and fluxes in rivers of the Tibetan Plateau.

Table S4. Annual precipitation, vegetation cover, concentrations of dissolved carbon and nitrogen in rivers of the Tibetan Plateau.

Table S1. Sampling information in rivers of the Tibetan Plateau.

	Sample date	Location		Elevation	Water-temp	pH
		Latitude (N)	Longitude (E)	m	℃	
Yellow River						
YL-1	8/17/2014	34°27′	97°57′	4372	15.1	8.29
YL-2	8/15/2014	34°53′	98°10′	4220	15.9	8.82
YL-3	8/18/2014	35°42′	99°33′	3780	16.2	8.60
YL-4	8/11/2014	37°02′	99°44′	3199	11	8.27
YL-5	8/11/2014	37°13′	100°29′	3279	11.5	8.52
YL-6	8/12/2014	36°54′	101°00′	3006	10.4	8.49
YL-7	8/12/2014	36°39′	101°33′	2346	14.3	8.48
YL-8	8/15/2014	35°00′	98°04′	4241	20.9	8.31
YL-9	8/18/2014	35°03′	98°42′	4475	8	8.36
YL-10	8/13/2014	36°03′	101°24′	2196	9.5	8.35
YL-11	8/19/2014	36°08′	103°37′	1525	15.6	8.29
Yangtze River						
YZ-1	8/16/2014	34°06′	97°38′	4701	13	8.44
YZ-2	8/17/2014	33°27′	97°17′	4294	16.6	8.51
YZ-3	8/16/2014	33°10′	97°22′	3953	11.4	8.42
YZ-4	8/16/2014	32°59′	97°15′	3521	13.1	8.28
Yarlung Tsangpo						
YT-1	5/10/2015	29°44′	83°59′	4587	13.3	8.67
YT-2	5/10/2015	29°18′	85°13′	4475	9.5	8.29
YT-3	5/9/2015	29°07′	87°35′	4004	11.3	8.34
YT-4	5/6/2015	29°20′	91°52′	3616	14.3	8.24
YT-5	5/25/2015	29°26′	94°30′	3131	17.5	
YT-6	8/25/2014	29°30′	94°25′	3118	9.5	
YT-7	5/9/2015	29°30′	86°21′	4715		
YT-8	5/10/2015	29°33′	84°57′	4575	9.8	
YT-9	5/9/2015	29°29′	86°02′	4852	6.5	
YT-10	5/9/2015	29°15′	88°55′	3827	10	8.81
YT-11	5/6/2015	29°23′	90°52′	3602	14.5	8.89
YT-12	9/3/2014	29°45′	94°44′	3351	16.2	
YT-13	8/28/2014	29°46′	94°45′	3332	10.7	
YT-14	8/28/2014	29°46′	94°45′	3330	8.3	
YT-15	8/25/2014	29°24′	94°26′	2898	12.3	
Indus						
ID-1	5/15/2015	32°31′	80°09′	4311	6.7	8.76
ID-2	5/15/2015	31°47′	80°17′	4440	6.5	8.43

Table S2. Estimated k (m d^{-1}) based on stream velocity (V, in m s^{-1}) and slope (S; unitless) for the sampling sites.

River catchment	Gauging station	Controlled sampling sites	location Lat. (N)	Lon. (E)	Elevation m	V (m/s)	S ‰	k m d^{-1}
Yangtze River	Tuotuo He	YZ-1	34°13′	92°27′	4527	0.9	1.67	6.5
	Zhimenda	YZ-2,3	33°02′	97°13′	3525	2.2	0.99	8.2
	Gangtuo	YZ-4	31°38′	98°35′	3034	1.7	1.12	7.5
Yellow River	Huangheyan	YL-1	34°53′	98°10′	4091	2.0	2.63	28.1
	Jimai	YL-2	33°46′	99°39′	3963	1.4	0.39	17.0
	Maq Qu	YL-3	33°58′	102°05′	3439	1.0	0.90	3.6
	Tangnaihai	YL-4-9	35°3′	100°09′	2686	2.2	2.02	4.5
	Guide	YL-10	36°02′	101°24′	2205	1.4	2.54	14.5
	Lanzhou	YL-11	36°04′	103°49′	1520	1.7	1.82	12.1
Yarlung	Lhase	YT-1-3,7	29°07′	87°37′	4477	1.4	4.45	20.0
Tsangpo	Nugesha	YT-4	29°20′	89°38′	3778	1.8	1.18	8.2
	Yangcun	YT-5	29°16′	91°48′	3549	1.9	1.18	8.4
	Nuxia	YT-6	29°28′	94°38′	2920	2.3	1.18	9.9
	Dogxung*	YT-8,9	29°25′	87°53′	3961	1.6	6.20	29.5
	Shigatze	YT-10	29°19′	88°52′	3837	1.2	6.09	22.8
	Lhasa	YT-11	29°38′	91°05′	3652	1.0	2.94	10.4
	Gongbujiangda	YT-12,15	25°93′	93°14′	3420	2.0	1.26	9.1
	Lulang*	YT-13	29°45′	94°44′	3332	2.0	2.26	14.9
	Lulang*	YT-14	29°45′	94°44′	3330	1.0	1.04	5.0
Indus	A'li*	ID-1,2	80°09′	32°30′	4311	1.2	4.25	16.9

Note: Data from the Hydrological Yearbook of China; * data from in situ observation.

Table S3. GHGs partial pressures and fluxes in rivers of the Tibetan Plateau.

	Stream order	pCO_2	pCH_4	pN_2O	CO_2 flux	CH_4 flux	N_2O flux
			μatm		g-C m^{-2} d^{-1}	g-C m^{-2} d^{-1}	g-N m^{-2} d^{-1}
Yellow River							
YL-1	1	1771	4.2	0.20	14260	24.8	0.10
YL-2	1	560	25.6	0.24	619	40.0	0.08
YL-3	2	856	4.6	0.25	1436	6.9	0.10
YL-4	2	971	9.4	0.26	6333	60.4	0.34
YL-5	2	873	7.8	0.25	5378	48.2	0.27
YL-6	2	982	16.6	0.28	6502	114.6	0.43
YL-7	2	950	10.5	0.56	5265	62.0	2.24
YL-8	3	974	3.5	0.26	4899	14.6	0.38
YL-9	3	1210	7.4	0.35	9822	50.9	1.45
YL-10	1	1140	8.4	0.31	6606	44.9	0.41
YL-11	1	1630	23.9	0.34	7588	111.4	0.32
Yangtze River							
YZ-1	3	959	4.8	0.22	2791	11.9	0.12
YZ-2	3	1051	5.1	0.26	3484	14.7	0.26
YZ-3	3	1244	14.8	0.26	5035	56.2	0.25
YZ-4	3	963	4.7	0.22	3068	12.6	0.05
Yarlung Tsangpo							
YT-1	3	382	2.3	0.19	1810	11.9	0.07
YT-2	3	489	0.6	0.16	3420	-4.5	-0.36
YT-3	3	566	0.3	0.11	3983	-8.1	-0.95
YT-4	3	545	0.4	0.12	1325	-2.9	-0.36
YT-5	3	392	2.0	0.28	510	2.7	0.20
YT-6	3	639	8.0	0.25	2377	35.0	0.15
YT-7	1	348	0.9	0.12	2357	-2.0	-1.06
YT-8	1	575	0.8	0.21	6685	-4.1	0.45
YT-9	2	361	0.6	0.09	3085	-6.0	-1.63
YT-10	2	539	8.8	0.12	4264	91.3	-1.07
YT-11	2	304	15.1	0.15	280	67.5	-0.31
YT-12	1	1257	200.5	0.29	4763	817.9	0.30
YT-13	1	963	10.5	0.48	6525	70.3	2.10
YT-14	1	980	14.8	0.32	2442	37.0	0.30
YT-15	1	591	7.7	0.25	1685	28.5	0.09
Indus							
ID-1	3	373	1.3	0.19	1702	2.2	-0.04
ID-2	3	430	1.3	0.22	2468	2.5	0.30

Table S4. Annual precipitation, concentrations of dissolved carbon and nitrogen in rivers of the Tibetan Plateau.

	DIC	DOC	DIN	DON	Prep-annual
	mg L^{-1}	mg L^{-1}	mg L^{-1}	mg L^{-1}	mm
Yellow River					
YL-4	34.77	1.98	0.70	0.06	326.41
YL-8	39.10	2.57	0.06	0.51	315.67
Yangtze River					
YZ-3	59.71	0.85	0.60	0.13	539.77
YZ-4	34.43	1.87	0.30	0.24	509.66
Yarlung Tsangpo					
YT-1	22.97	2.29	0.15	0.12	416.58
YT-2	28.04	1.66	0.19	0.07	532.83
YT-3	19.94	1.04	0.22	0.11	327.32
YT-4	21.23	0.91	0.33	0.22	410.91
Indus					
ID-1	29.13	0.85	0.24	0.24	144.49
ID-2	19.99	1.13	0.28	0.46	75.39

References

Hall Jr, R.O., Tank, J.L., Baker, M.A., Rosi-Marshall, E.J., Hotchkiss, E.R. 2016 Metabolism, gas exchange, and carbon spiraling in rivers. Ecosystems 19, 73-86.

Raymond, P.A., Zappa, C.J., Butman, D., Bott, T.L., Potter, J., Mulholland, P., Laursen, A.E., McDowell, W.H., Newbold, D. 2012 Scaling the gas transfer velocity and hydraulic geometry in streams and small rivers. Limnology & Oceanography: Fluids & Environments 2, 41-53.
